# An Inclusive Entrepreneurial Path Model Based on Rural Digital Entrepreneurship Data in Zhejiang Province Using Few-Shot Learning

**DOI:** 10.1155/2022/8015681

**Published:** 2022-05-09

**Authors:** Xiangmin Meng, Jie Zhang, Min Sun

**Affiliations:** ^1^College of Economics and Management, Nanjing University of Aeronautics and Astronautics, 29 Jiangjun Avenue, Nanjing, Jiangsu 211100, China; ^2^Zhejiang Wanli University, 8 Qianhu South Road, Ningbo, Zhejiang 315100, China

## Abstract

The main strategic direction of promoting rural revitalization is to achieve high-quality rural development, maintain the dominant position of farmers, and promote common prosperity of farmers. In recent years, the sustainable development of economy has raised farmers' awareness of starting their own businesses, and their entrepreneurial enthusiasm has been heating up. Based on the empirical survey data of typical rural areas in the Zhejiang Province and structural equation model analysis technology, this paper makes an empirical analysis of the relationship among network embeddedness, entrepreneurial resources, and entrepreneurial ability. The results show that knowledge resources have a significant negative impact on farmers' inclusive entrepreneurial behavior. The dual embedment of social network and industrial network has a significant positive impact on migrant workers' access to knowledge resources and operational resources needed for entrepreneurship, and the entrepreneurial ability of migrant workers mainly depends on operational resources. Under the background of common prosperity, we should continue to implement the policy of supporting farmers' inclusive entrepreneurship and encouraging industrial and commercial capital to enter rural areas, and at the same time, provide rural areas with agricultural knowledge, agricultural skills, and market guidance to promote employment.

## 1. Introduction

In China's “new normal” stage, entrepreneurship is one of the most important ways to promote the stable operation of the economic system and long-term economic development. However, as there is a common threshold for capital entry to participate in entrepreneurial innovation, the credit exclusion of countries and regions has become a key factor affecting the entrepreneurial activities of small- and medium-sized enterprises and ordinary families. Inclusive development combines economic development with the needs of vulnerable groups, putting people first and achieving social justice and fairness [1, 2]. Since then, “inclusive development” has supplanted “inclusive growth” as the compass for the new era's detailed economic development [3]. Inclusive innovation is defined as a type of innovation that promotes inclusive development, “reduces poverty,” and it is “most directly related to the needs of the poor.” The will, motivation, and vitality of rural entrepreneurship have been boosted to unprecedented levels by the Common Prosperity Strategy, and more rural entrepreneurs have emerged as the protagonists of poverty alleviation, industrial integration, social governance, and rural areas [4]. As a result, tapping the rural social network and understanding the impact of network embedding on the acquisition of entrepreneurial resources and the improvement of farmers' entrepreneurial ability is critical for farmers' employment and inclusive entrepreneurship.

Farmers' self-employment consciousness gradually emerges as the economy continues to develop. The development of this self-employment consciousness has a more positive impact on farmers' income and employment prospects. Farmers' needs for resources and employment methods in the process of starting their own businesses are also posing a challenge to China's original urban-rural dual system [5]. The revitalization of farmers and rural areas is an important part of common prosperity in the traditional concept, as well as the focus and difficulty of common prosperity [6]. According to Ma and others, China's rural governance should be focused on resolving interdependence and cooperation among people, resolving contradictions and conflicts, and improving people's public services [7]. In academic circles, most small-scale entrepreneurship research focuses on regional differences in small-scale entrepreneurs, as well as the impact of training and entrepreneurial intent on small-scale entrepreneurship. The government's role is to make timely and effective institutional arrangements to create and improve entrepreneurial conditions, optimize the entrepreneurial environment of microsubjects, and improve the overall quality of the entrepreneurial environment. Creating a strong entrepreneurial atmosphere and mature entrepreneurial conditions is the goal of creating an entrepreneurial environment. This paper makes some recommendations to the government based on the research on inclusive entrepreneurial paths to improve the overall entrepreneurial environment, promote the popularization of typical inclusive entrepreneurial paths, and improve the quality of rural entrepreneurial activities.

Highly developed social wealth and social productivity are the premise and foundation of common prosperity. It is difficult to achieve common prosperity unless productivity is highly developed. Despite the fact that farmers' wages and operating income have increased, the income structure still needs to be optimized. Wages and operating income are excessively high, and property income is also rising, however, it is rising only as a small percentage of total income. Supporting the entrepreneurial environment, encouraging farmers to start their own businesses, and focusing on optimizing the rural entrepreneurial environment are the keys to success. This paper can help farmers learn from the research results, obtain the most effective entrepreneurial resources in limited circumstances, maximize their abilities, and complete business creation with quality and policy support factors by studying and analyzing the impact of resource acquisition on farmers' inclusive entrepreneurial path.

The innovations of this paper are as follows:

1. Innovation from the perspective of research: the evolution of entrepreneurial enterprises has its own life cycle. This paper examines a comprehensive entrepreneurial path based on the life cycle of entrepreneurial enterprises, i.e., selecting existing research from the perspective of enterprise life cycle will gain more targeted characteristics of inclusive entrepreneurial path.
2. Innovation of research content: the existing research literature on farmers' entrepreneurship mainly focuses on the entrepreneurial environment, and the research on farmers' entrepreneurial access to resources is insufficient. Based on the household survey of typical farmers in the Zhejiang Province, this paper collected the data needed for the research. The form of investigation solves the limitation of time and space well. The investigation can be conducted in different places at the same time, and the quantity and quality of samples can be guaranteed using network tools.

The research framework of this paper consists of five parts, which are arranged as follows: the first chapter introduces the research background and significance, and then, it introduces the main work of this paper. The second chapter mainly introduces the related research status of inclusive entrepreneurship. The third chapter puts forward the concrete method and implementation of this research. The fourth chapter verifies the superiority and feasibility of this research model. The fifth chapter is the summary and prospect of the full text.

## 2. Related Work

### 2.1. Inclusive Entrepreneurship Research

Individuals who are entrepreneurs start businesses in one of the two ways: as new ventures or as derivatives of existing ventures. Early academic circles believed that the success or failure of entrepreneurs was determined by their personal characteristics and talents, however, later research has shown that it is the position of individuals in the organization, not the characteristics of entrepreneurs, that determines whether they seek opportunities and engage in entrepreneurial activities [8]. As a result, situational factors and mechanisms become the main ideas to explain entrepreneurial activities in the process of entrepreneurship, which is also the theoretical framework on which this paper is built, i.e., it examines the key features of the overall entrepreneurial process before moving on to the background factors that may influence entrepreneurial activities.

Yu and others believe that inclusive entrepreneurship is actually a collective entrepreneurial activity carried out by ordinary grassroots people in specific areas, and it is a self-employed group [9]. Essougong and others believe that a key feature of inclusive entrepreneurship is the realization of equal opportunities. Inclusive entrepreneurial opportunities are different from entrepreneurial opportunities in general sense. Opportunities appear in specific areas and are strongly influenced by background factors, such as regional cluster environment and government [10]. Oladele et al. think that natural conditions, history, accidental factors, economies of scale and externalities, enterprise organizational structure, competition, and innovation are the key factors for the formation of industrial clusters [11]. Deller et al. established the indicator system of inclusive development and found that inclusive development had an indirect impact on urban-rural income gap using the intergenerational overlap model [12]. From the perspective of microfamily, Ab et al. established a multivalue response model and analyzed inclusive finance from the supply side and demand side [13]. According to the empirical results, the optimal level of inclusive finance is not necessarily the same as the level of demand and supply and is also different from the macrolevel and microlevel. Nie et al. conducted a study based on panel data to further confirm the importance of entrepreneurship and policy support to long-term economic growth [14]. Therefore, although work experience has a positive impact on entrepreneurship, the influence of personal education and the family background of public officials on entrepreneurship is uncertain. Empirical research shows that households with high risk aversion are more inclined to choose agricultural entrepreneurship, households with risk preference are more inclined to start business in industry and commerce, and the income of industrial and commercial entrepreneurial enterprises shows a reciprocal U-shaped relationship.

### 2.2. Farmers' Entrepreneurship

In the process of farmers' entrepreneurship, farmers rely on family organizations (or informal organizations composed of relatives and friends) or create new organizational forms to form large-scale production and operation scale and pursue the growth and development of wealth. Zhao et al. divided small-scale entrepreneurship into group entrepreneurship and individual entrepreneurship and pointed out that the content of small-scale entrepreneurship referred to the expansion of processing agriculture, and so on [15]. Liu et al. regarded farmers' entrepreneurship as an unbalanced game process that involved the transformation of various resources, and it is also the decisive factor of policy and market in the game [16]. In terms of the influence of farmers' entrepreneurial behavior on society and economy, Phiri et al. explained the importance of farmers' entrepreneurial behavior to rural development [17]. Zhang and Han also made clear the strategic significance of farmers' entrepreneurial actions to the harmonious development of new countryside [18].

More academics are looking at the growth of migrant workers' entrepreneurship through the lens of the entrepreneurial network. The importance of enterprise growth includes scale expansion and capability improvement, and entrepreneurship can be divided into social networks and industrial networks. Shah et al. discovered that migrant workers who engage in growth-oriented and value-oriented entrepreneurship outperform migrant workers who engage in survival-oriented entrepreneurship, implying that higher value pursuit yields better results [19]. The number of families reduces the likelihood of migrant workers returning home to start a business, and the accumulation of family wealth (economic and social capital) has a positive impact on entrepreneurial performance. Sher et al. believe that because they have more knowledge and skills, better education and training, stronger adaptability to the environment, higher self-pursuit, and the new generation of farm tools, they are more willing to return home and start their own businesses [20]. Apart from the difficulty of policy factors, Blevins and Ragozzino have shown that human capital and the individual psychological state of entrepreneurs have a significant impact on farmers' entrepreneurial performance [21]. According to Gans et al., if a company's information capability is low, it will lack the necessary market information and information processing mechanisms [22].

## 3. Methodology

### 3.1. Theoretical Model

Entrepreneurship is a form of work that necessitates the development of management, organizational, and service skills, as well as the ability to think, reason, and make decisions. Entrepreneurship is a branch of business that studies how individuals create, discover, or create opportunities to create new things, and how they use those opportunities in various ways to produce different outcomes. With the continued development of our economy and society, an increasing number of people will join the middle class. Many farmers will be present, particularly those who are most likely to enter the middle class. To achieve universal prosperity, we must not only make rural residents the primary beneficiaries of common prosperity but also assist them in achieving material and spiritual prosperity.

Entrepreneurial motivation is the goal or vision that entrepreneurs demonstrate when starting a business based on their internal and external needs. It drives entrepreneurial behavior and influences entrepreneurial performance during the startup process. Regardless of the aforementioned influences on entrepreneurship, entrepreneurs decide whether to start a business based on their own internal needs. Furthermore, outstanding employees from successful peer companies frequently start their own businesses. They are well-versed in the industry's operations and are well-versed in the most critical aspects of the industry's operations. They can grasp the important points when starting a business and achieve twice the result with half the effort, lowering the cost of the enterprise's startup period. Entrepreneurs are well aware of the flaws and drawbacks of running a business, and they can avoid the risks of starting one as soon as possible.

**Figure 1:**
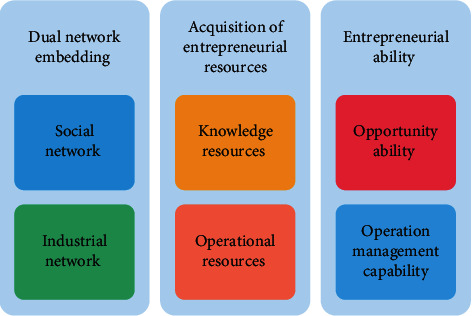
Theoretical model of farmers' inclusive entrepreneurial ability.

However, unlike the inherent characteristics of other entrepreneurial groups' networks, migrant workers fall into the predicament of leaving the social and industrial networks in the process of entrepreneurship. As they are far away from their hometown, they are hard to integrate into the city, forming a social network, while migrant workers who return home to start their own businesses are far away from the city. Hence, it is difficult for them to obtain external economy in terms of industrial division of labor and cooperation through participation. The purpose of this study is to explore the influence path of dual networks embedded in farmers' inclusive entrepreneurial ability on the acquisition of entrepreneurial resources. Based on this, this paper puts forward the theoretical model as shown in Figure 1.

Entrepreneurship is the process of creating new products or services and realizing their potential value in the absence of resources. Except for the donation of entrepreneurial resources and entrepreneurial opportunities, entrepreneurs often have nothing. There is a “resource gap” between the huge number of resources needed for starting a business and the limited resources actually controlled by new enterprises. At the initial stage of entrepreneurship, the inherent resource holding of entrepreneurs in the entrepreneurial environment is the basis of entrepreneurial behavior, and the heterogeneity of resource holding composition determines the randomness and diversity of entrepreneurial behavior at the externalized microlevel. With the new creation of some entrepreneurs, the performance of enterprises is obviously better than that of other entrepreneurs. At this stage, entrepreneurs should have the following related skills: integrate resources and start and operate enterprises.

### 3.2. Life Cycle Model of Enterprise

The age of a company is the basis of its life cycle, which is mainly reflected in the relationship between two factors: adaptability and controllability. Adaptability refers to the response speed and controllability of enterprises to changes in internal and external environment. Controllability refers to the ability of a company to control its external and internal environment. The “youth” of an enterprise refers to its adaptability, which is relatively easy to change and adjust, however, its control is relatively small, and its behavior is generally unpredictable. Therefore, adaptability and controllability are two main factors. The growth of enterprises is the common growth of quality and quantity. Any model that only explains the growth of a company in terms of quality or quantity is one-sided and incomplete. In the three-dimensional enterprise life cycle model, the company life cycle is determined by the age and scale of the company. This model not only reflects the quality of enterprises but also reflects the change of enterprise scale. Hence, it can describe the growth trajectory of enterprises more comprehensively.

**Figure 2:**
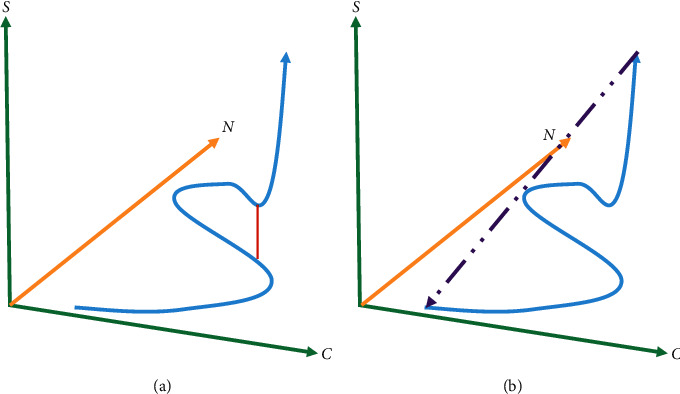
Enterprise growth model based on the (a) Ivy growth model and the (b) Meteor pattern.

A typical enterprise life cycle refers to the whole continuous process of an enterprise from birth to growth, aging, and final decline, however, the enterprise is not an organism. Hence, we cannot apply the biological point of view. Theoretically speaking, it is meaningful to study the growth model of a company.  Figure 2 shows the growth model of enterprises in the two models.

For a successful small company, it generally adopts the Ivy enterprise development model. A company can maintain adaptability and flexibility until its heyday. The company can maintain stable growth while extending its scale if it can achieve controllable equilibrium and keep up with the changing market (Figure 2(a)). There is also a meteor model. When a company's life develops to infancy or adolescence, the scale of the company expands rapidly and then suddenly dies, and the growth curve is like the trajectory of a meteorite falling from the sky (Figure 2(b)).

Every stage of the life cycle is very important. Enterprises have different strategic priorities at different stages, and failure at any stage will destroy entrepreneurial enterprises. During the growth of a new enterprise, there may be several problems one after another. Therefore, we need the influx of entrepreneurs, teams, resources, and other factors to constantly overcome various difficulties in the growth process of entrepreneurial enterprises. Entrepreneurs should constantly adapt to the environment, adjust team members, integrate accumulated resources, and improve the overall quality.

### 3.3. Data Source

**Figure 3:**
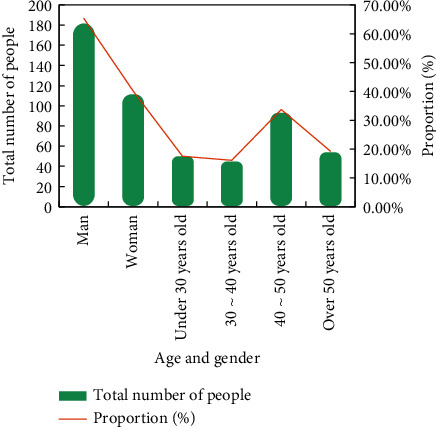
Basic information of sample farmer entrepreneurs.

The data of this paper comes from the survey of farmers' entrepreneurship and employment in the Zhejiang Province. In terms of regional selection, the survey samples are selected from farmer entrepreneurs in the typical villages of the Zhejiang Province. The questionnaire is distributed in-person and is filled on the spot. A total of 300 questionnaires were distributed and 285 were returned, with an effective rate of 95%. The contents of the survey mainly include personal and family information of farmers, characteristics of farmers' entrepreneurial environment, characteristics of farmers' entrepreneurial behavior, etc. When studying the personal information and family information of farmers, the most important thing is whether they will communicate with public officials and have good relations with state-owned banks and other government agencies to determine whether there are key factors for obtaining resources from resource acquisition. The main gender and age of entrepreneurs are shown in Figure 3.

### 3.4. Mathematical Model Establishment and Variable Selection

This paper aims to investigate how migrant workers' inclusive entrepreneurial activities can acquire the necessary knowledge and management resources for entrepreneurship via the dual embedding of social and industrial networks, as well as influence farmers' inclusive entrepreneurial ability. The structural equation model (SEM) is a multivariate statistical technique that combines factor and path analysis. It can incorporate some difficult-to-measure variables directly into the model for analysis, as well as analyze the complex relationship between multiple observed variables and latent variables all at once, which traditional regression analysis cannot. The structural model's formula is as follows:

(1) $$\begin{array}{c} X=\Gamma_{X}\xi +\sigma , \end{array}$$

(2) $$\begin{array}{c} Y=\Gamma_{Y}\tau +\varepsilon . \end{array}$$

 Equations (1) and (2) are measurement models. Among them, *X*, *Y* represent exogenous and endogenous observed variables, respectively. *ξ*, *τ*, respectively, represent exogenous latent variables (i.e., potential independent variables) and endogenous latent variables (i.e., potential dependent variables). Γ_*X*_ is the relationship matrix between the exogenous latent variable and its observed variable, which is composed of a factor load of *X* on *ξ*. Γ_*Y*_ is the relationship matrix between the endogenous latent variables and their observed variables, which is composed of the factor load of *Y* on *τ*. *σ*, *ε* is the error term of the measurement model.

(3) $$\begin{array}{c} \tau =A\tau +\Lambda \xi +\gamma . \end{array}$$

 Formula (3) is a structural model in which *A* is the coefficient matrix of the endogenous latent variables, describing the relationship between endogenous latent variables, Λ is the coefficient matrix of exogenous latent variable, describing the influence of exogenous latent variable *ξ* on endogenous latent variable *τ*, and *γ* is the error term of the structure.

Farmers' entrepreneurial behavior has two possibilities: starting a business and not starting a business. However, in the existing regression model, the value range of dependent variable is between negative infinity and positive infinity, while the value range of the dependent variable affected by entrepreneurial behavior is within [0, 1]. The form of binary dependent variable model is as follows:

(4) $$\begin{array}{c} {\hat{y}}_{1}=x_{i}\beta +\mu_{i}, \end{array}$$

where *μ*_*i*_ is the interference term.

Assuming that ${\hat{y}}_{1}$ is greater than the critical value of 0, *y*_*i*_=1. When ${\hat{y}}_{1}\leq 0$, *y*_*i*_=0. The relationship is as follows:

(5) $$\begin{array}{c} y_{i}=\left\{\begin{array}{cc} 1 & {\hat{y}}_{1}>0,\\ 0 & {\hat{y}}_{1}\leq 0. \end{array}\right. \end{array}$$

 Here, the critical value is chosen as 0. In fact, as long as *x*_*i*_ contains a constant term, the critical value is irrelevant. Hence, at this point,

(6) $$\begin{array}{c} P\left(y_{i}=1|x_{i},\beta \right)=P\left({\hat{y}}_{1}>0\right)=P\left(\mu_{i}>-x,\beta \right)=1-F\left(-x,\beta \right),\\ P\left(y_{i}=0|x_{i},\beta \right)=P\left({\hat{y}}_{1}\leq 0\right)=P\left(\mu_{i}\leq -x,\beta \right)=F\left(-x,\beta \right), \end{array}$$

where *F* is the distribution function of *μ*_*i*_, which requires that it is a continuous function and monotonically increasing. Hence, the regression model can also be regarded as follows:

(7) $$\begin{array}{c} y_{i}=1-F\left(-x,\beta \right)+\mu_{i}. \end{array}$$

At this point, it becomes the mean regression model of *y*_*i*_.

112 data obtained from the questionnaire were used as expert opinions. Establish fuzzy number $\tilde{A}$.

(8) $$\begin{array}{c} \tilde{A}=\left(\alpha_{i},\beta_{i},\gamma_{i}\right),\\ \alpha_{i}=\min\left(B_{ij}\right),\\ \beta_{i}={\left(\prod_{k=1}^{n}B_{ij}\right)}^{1/n},\\ \gamma_{i}=\max\left(B_{ij}\right). \end{array}$$

 In the above formula: *i*—Index number, *i*=1,2, ⋯, *n*; *j*—Expert number, *i*=1,2, ⋯, *n*; *α*_*i*_—The minimum value of index *i* scored by experts; *β*_*i*_—Geometric mean value of expert scoring index *i*; *γ*_*i*_—The maximum value of expert's score on index *i*; *B*_*ij*_—*j* expert's scoring value of index *i*.

When using the fuzzy Delphi method to evaluate risk indicators, we need to convert linguistic variables into fuzzy numbers. Hence, we import expert scoring data into our model and get triangular fuzzy numbers. The fuzzy number is obtained by inverse clearing triangular fuzzy number by the arithmetic average method.

(9) $$\begin{array}{c} A=\frac{\alpha_{i}+\beta_{i}+\gamma_{i}}{3}. \end{array}$$

**Figure 4:**
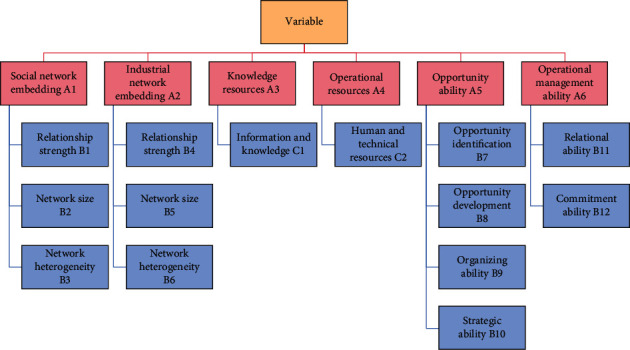
Variable measurement item.

In this study, to ensure the validity and reliability of the measurement indicators, some items were modified and supplemented according to the characteristics and actual situation of farmers' inclusive entrepreneurs using the maturity scale used in existing domestic and foreign literatures. The questionnaire was designed by Likert's 5-point scoring method, and the description of the items was described as “strongly agreed,” “comparatively agreed,” “moderate,” “slightly disagreed,” and “very different,” as shown in Figure 4.

#### 3.4.1. Dual Network Embedding

As migrant workers start their own businesses mainly in industries with low skill content, it is difficult for new enterprises to have the power to establish cooperative relations with universities and research institutes. It represents a group of related partnerships among subsidiaries, financial institutions, intermediaries, and government agencies, excluding universities and scientific research institutions.

#### 3.4.2. Entrepreneurial Resources

This paper investigates the acquisition of knowledge resources by farmers' inclusive entrepreneurs from four aspects: new product or service development, market development, production and management, and related policy system.

#### 3.4.3. Operating Resources

The existing research mainly inspects business resources from the aspect of resource availability, focusing on whether entrepreneurs can obtain capital, human resources, technical resources, plant, equipment, and other physical resources at low cost.

#### 3.4.4. Entrepreneurial Ability

This paper describes entrepreneurs' management ability from four dimensions: organizational ability, strategic ability, relationship ability, and commitment ability. Farmers' inclusive entrepreneurs should have the same relationship and commitment skills as other entrepreneurial groups, however, their newly started enterprises are small in scale and simple in organization, and their requirements for organizational and strategic capabilities are slightly lower than those of other entrepreneurs.

## 4. Experiment and Results

The estimated coefficients in the binary choice model cannot be interpreted as the marginal effect of symbols on dependent variables only. If the sign is evidence, the probability that the dependent variable is 1 increases. If the sign is negative, the probability that the dependent variable is 1 decreases. Considering that the dependent variable is the implementation of farmers' entrepreneurial behavior in variable selection, i.e., the dependent variable can take the values of “1” and “0” as “entrepreneurship” and “for entrepreneurship.” In this study, resource acquisition mainly starts with policy resources, financial resources, and information resources. Hence, the selection of variables is shown in Table 1.

**Figure 5:**
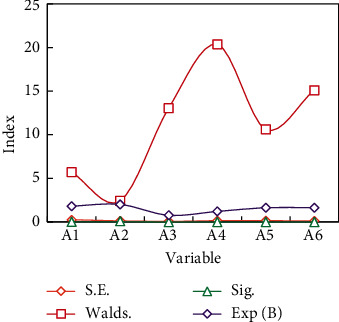
Influencing factors of various variables.

SPSS software is used to analyze the influence of resource acquisition on farmers' inclusive entrepreneurial behavior, and the regression results are shown in Figure 5.

*A*4 has a significant positive correlation with farmers' entrepreneurial behavior of acquiring information resources, which is significant at the statistical level of 1%. The regression coefficient is 0.196 and Exp(*B*) is 1.21, which means that the probability of farmers' entrepreneurial behavior will increase by 0.196 for each additional unit in contact with civil servants, other things being equal. At the level of 5%, *A*1 has a significant impact on farmers' entrepreneurial behavior, with the coefficient of 0.569 and Exp(*B*) of 1.806, which means that the probability of farmers' entrepreneurial behavior will increase with each unit of *A*1. Therefore, the probability of entrepreneurial behavior of married farmers is higher than that of unmarried farmers.

**Figure 6:**
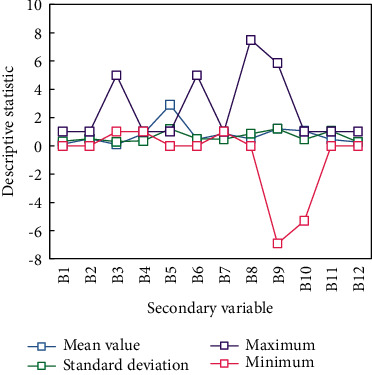
Descriptive statistics of related variables of secondary variables.

*A*3 has a significant negative impact on farmers' entrepreneurial behavior. It passed the 1% significance test, and the coefficient was −0.215 and Exp(*B*) was 0.774. It means that the higher the education level of farmers, the lower the possibility of entrepreneurial action. Studies have shown that industrial structure adjustment will affect regional innovation activities [23]. The research shows that the optimization and upgrading of industrial structure has a positive effect on inclusive entrepreneurship [16]. Research shows that the rapid change of industrial structure will affect entrepreneurship in the short term, however, it will promote local entrepreneurial innovation activities in the long-term [20]. Therefore, this paper selects the proportion of the tertiary industry in GDP to investigate to control the industrial structure of each region. Descriptive statistics of each variable are shown in Figure 6.

The scale design of this paper refers to the mature scale used by scholars in the past, which ensures the validity of the content of this scale. Constructive validity, also known as structural validity, is mainly used to indicate whether the actual measurement result of a scale is consistent with the research concept, which usually consists of convergence validity and discrimination validity. Specifically, *A*1 and *A*2 can extract three factors, *A*3 and *A*4 can extract a single factor, *A*5 can extract two factors, and *A*6 can extract four factors. These extracted factors are measured by a scale, and the variables are the same. At the same time, the factor loading values of all measures after orthogonal rotation are greater than the threshold value of 0.5, which indicates that the convergence validity of the measure scale is ideal. The discriminant validity test results (see Table 2) show that the square root of latent variables on the diagonal is larger than the correlation coefficient between latent variables on off-diagonal, which indicates that the scale has good discriminant validity.

**Figure 7:**
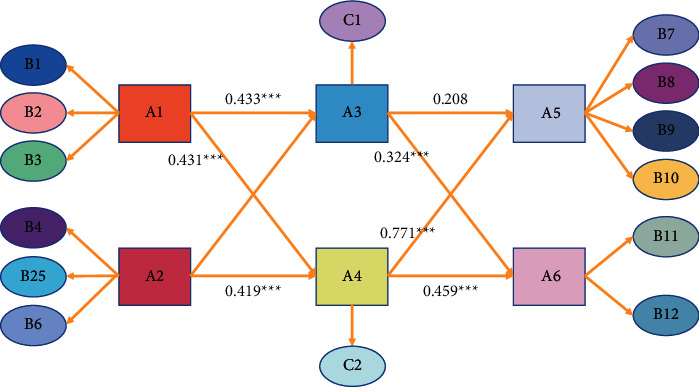
Structural equation model diagram of the influence of farmers' inclusive entrepreneurship.

The result of building structural equation model with AMOS is shown in Figure 7. All the test indexes of the overall goodness-of-fit of the model are higher than the threshold, which indicates that the overall goodness-of-fit of the model is good.

In this paper, SPSS20.0 statistical software is used to analyze the impact of resource acquisition on farmers' business performance. In the process of processing, firstly, all variables are introduced into the regression equation. Then, the significance test is carried out, and finally, the regression method is refitted, and this principle is repeated until the regression coefficient of each variable in the equation is significant.

**Figure 8:**
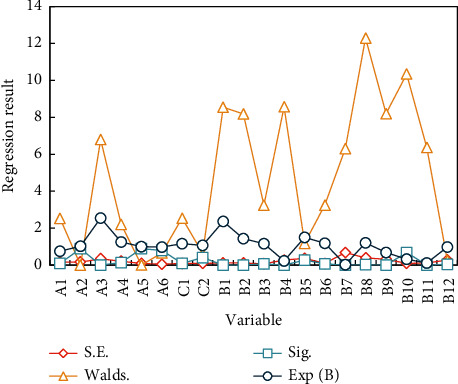
The first step model regression results.

We introduce all the above independent variables into the regression equation, as shown in Figure 8 below.

**Figure 9:**
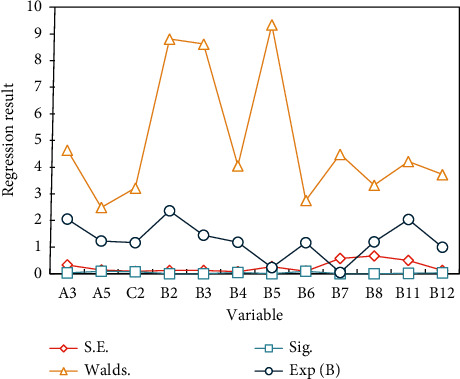
The third step model regression results.

It can be seen in *A*2, *A*4 and *A*6, *B*1 that it is not obvious. After removing the above variables, a two-step regression analysis is introduced, which shows that insignificant variables are equal to *C*1 and *B*10. So far, in the third step, we used two variables *A*1 and *B*9 and brought all the remaining variables into the regression analysis method to continue the regression analysis. The regression results of the third stage model are shown in Figure 9.

It is significant with *B*3 at the level of 5% in the statistical test, and the estimation coefficient is positive. When other factors remain constant, contact with senior civil servants improves the effectiveness of farmers' entrepreneurial performance. The difficulty level of high government function, relevant market system establishment, market policy guidance, financing convenience, contact with bank staff, access to information from other channels, overall access to information resources, policy convenience, and no resources is significant and failed the test.

Farmers' entrepreneurs in China are clearly affected by information restrictions in the process of starting a business because of the limited information channels available to them and the high cost of obtaining information. Business operations and maintenance are no longer limited by information channels. The educational level is statistically significant at 10%. Information channels are becoming more diversified and efficient. The estimated coefficients of age and educational background are positive among the three variables, while the estimated coefficient of gender is negative. Farmers' entrepreneurial performance is significantly influenced by their representative life experience and accumulation of experience. Although operational resources serve a greater purpose than knowledge resources, both have a negligible impact. This result demonstrates that current migrant worker entrepreneurship is still survival-oriented, and the industry entry threshold is low. Inclusive entrepreneurs are relatively simple, and they have not yet attained the level of knowledge acquisition resources and operational resources required to improve a company's ability to operate and manage. At the moment, Chinese migrant workers are still self-employed, and their new entrepreneurial management mode differs significantly from the modern enterprise management mode that reflects migrant workers' entrepreneurial spirit.

## 5. Conclusions

Based on the empirical research data of typical villages in the Zhejiang Province, this paper analyzes and summarizes the process and mechanism of inclusive entrepreneurs' development path from the perspective of common prosperity. We find that there is a significant positive correlation between business resources and farmers' entrepreneurial behavior in obtaining information resources. At the statistical level of 1%, knowledge resources have a significant negative impact on farmers' entrepreneurial behavior. The dual embedment of social network and industrial network has a positive impact on migrant workers' acquisition of knowledge resources and business resources needed for starting a business, however, the acquisition of knowledge resources is mainly influenced by the embedded industrial network. Therefore, under the vision of common prosperity, when building various systems, the government fully considers the needs of people of different classes and actively introduces policies to encourage and guide farmers so that more farmers can choose entrepreneurship, employment, and settlement.

## Figures and Tables

**Table 1:** Selection of variables influencing access to resources on farmers' inclusive entrepreneurial path.

First-order variable	Secondary variable	Mean value
*A*1	*B*1	3.36
	*B*2	2.25
	*B*3	3.04
*A*2	*B*4	2.21
	*B*5	3.88
	*B*6	2.96
*A*3	*C*1	2.36
*A*4	*C*2	3.32
*A*5	*B*7	3.01
	*B*8	2.88
	*B*9	3.63
	*B*10	2.41
*A*6	*B*11	2.88
	*B*12	3.71

**Table 2:** Test results of discrimination validity.

Variable	*A*1	*A*2	*A*3	*A*4	*A*5	*A*6
*A*1	0.771					
*A*2	0.413	0.762				
*A*3	0.501	0.501	0.821			
*A*4	0.263	0.336	0.461	0.743		
*A*5	0.459	0.428	0.502	0.418	0.874	
*A*6	0.337	0.367	0.424	0.442	0.426	0.769

## Data Availability

The dataset used to support the findings of this study are available from the corresponding author upon request.
